# Integrated Doppler and Elastography Assessment of Hidradenitis Suppurativa and Dactylitis

**DOI:** 10.3390/diagnostics16071059

**Published:** 2026-04-01

**Authors:** José Alexandre Mendonça, Bárbara Brunca, Ana Paula Weber, Paula Tavares Colpas

**Affiliations:** 1 Stricto Sensu Postgraduate Program in Health Sciences/Ultrasonography/Rheumatology Service, Pontifical Catholic University of Campinas (PUC-Campinas), São Paulo 13060-904, Brazil; 2 Dermatology Service, Pontifical Catholic University of Campinas (PUC-Campinas), São Paulo 13060-904, Brazil; barbarabrunca@gmail.com (B.B.); anapaulaweber21@gmail.com (A.P.W.); paulacolpas@gmail.com (P.T.C.)

**Keywords:** hidradenitis suppurativa, dactylitis, musculoskeletal ultrasound, ultrasound elastography, strain elastography, microvascular Doppler, enthesitis, inflammatory angiogenesis, imaging biomarkers

## Abstract

Multimodal high-resolution ultrasound, including B-mode, Power Doppler, MicroVessel Doppler, spectral Doppler, and strain elastography, was used to assess concomitant dactylitis and hidradenitis suppurativa (HS) in a 46-year-old woman with severe hidradenitis suppurativa (IHS4 = 28), who was diagnosed 1.5 years ago and has been using adalimumab. Axillary ultrasound demonstrated abscess cavities and draining fistulous tracts with marked structural distortion, increased vascular signal on advanced Doppler modalities, and heterogeneous stiffness patterns on elastography, consistent with active deep inflammatory involvement. Simultaneously, evaluation of the third right finger revealed flexor tendon sheath thickening, soft-tissue edema, Doppler-positive inflammatory activity, and altered biomechanical properties compatible with dactylitis. High-resolution ultrasound has been increasingly recognized as a valuable tool for evaluating inflammatory and structural changes in cutaneous diseases, including HS. These multimodal findings illustrate how structural, vascular, and biomechanical ultrasound parameters may provide complementary information for characterizing inflammatory tissue remodeling in HS associated with dactylitis. As this report describes a single patient, these elastographic observations should be considered exploratory and hypothesis-generating rather than evidence of clinical validation.

**Figure 1:**
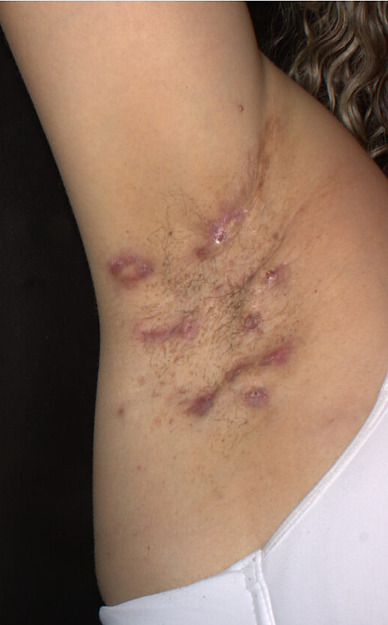
Axillary HS showing active inflammatory disease. Multiple deep-seated inflammatory nodules and abscesses are observed in the left axillary region, associated with interconnected draining sinus tracts and fibrotic scarring. The coexistence of nodules, abscesses, and draining tunnels is consistent with high inflammatory burden and advanced structural remodeling, corresponding to severe disease according to IHS4 [1].

**Figure 2:**
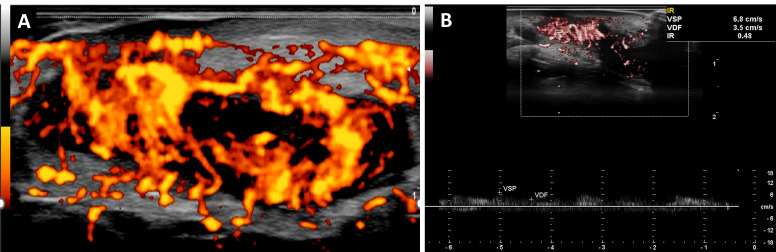
(**A**) High-resolution Power Doppler ultrasound (12.5 MHz; PRF 480 Hz) demonstrates a hypoechoic dermo-subcutaneous inflammatory complex corresponding to an abscess cavity and associated tunnel formation. Doppler settings were optimized for low-velocity flow detection, revealing dense intralesional and perilesional vascular signals. The marked hypervascularization reflects active inflammatory angiogenesis and microvascular remodeling, consistent with validated Doppler-based inflammatory biomarkers in HS [2,3,4,5]. (**B**) Spectral Doppler interrogation of an intralesional vessel demonstrates a low-resistance arterial waveform with a resistive index (RI) of 0.48, supporting active inflammatory hyperemia and neovascular activity. The low RI pattern is consistent with inflammatory-driven vascular proliferation rather than fibrotic or quiescent tissue. These findings reinforce the role of Doppler ultrasound as a noninvasive biomarker of disease activity and vascular dynamics in HS [6]. The same Doppler acquisition parameters were applied for the Doppler images shown in Figure 4 and Figure 6.

**Figure 3:**
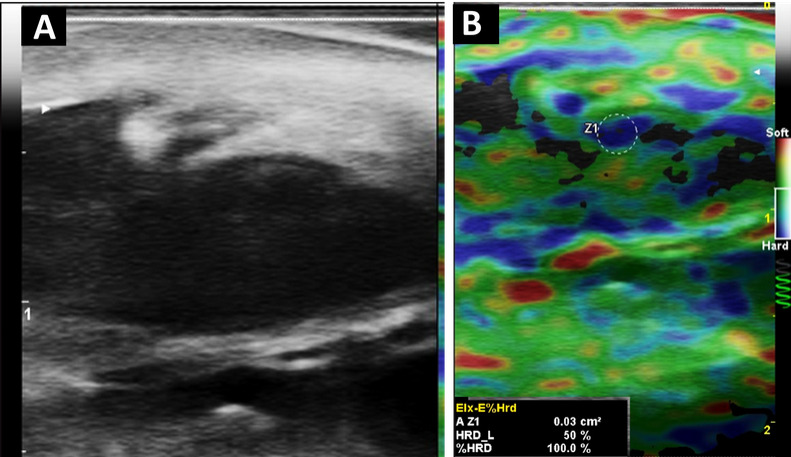
Structural and biomechanical assessment of the same HS lesion shown in Figure 2. High-frequency ultrasound was performed using a MyLab™ Omega system (Esaote S.p.A., Genoa, Italy; eXP platform with ElaXto strain elastography software) equipped with a 6–19 MHz linear transducer. (**A**) B-mode ultrasound of the axillary lesion demonstrates a hypoechoic dermo-subcutaneous cavity compatible with an abscess associated with a sinus tract, with architectural distortion of the dermal–subdermal layers. (**B**) Strain elastography reveals heterogeneous stiffness distribution within the abscess and tract wall. Elastography was acquired using light repetitive manual compression under real-time quality control (spiral indicator). Three consecutive compression cycles were performed, and measurements were obtained from four regions of interest (ROIs; 0.03 cm^2^ each), including the tract wall (Z1–Z2) and adjacent clinically unaffected reference tissue (Z3–Z4) for internal comparison at comparable depths within the elastographic field, with ROI placement guided by the corresponding B-mode anatomical reference and avoiding inclusion of fluid components or acoustic artifacts. The altered tract wall demonstrated a hard component ranging from 99.9% to 100% (%HRD), whereas adjacent reference tissue without a lesion showed substantially lower stiffness values (e.g., 9.8–13.9% HRD). Strain ratios (Elx2/1, Elx3/1, Elx4/1) ranged from 1.54 to 2.45, indicating relative biomechanical heterogeneity. These findings illustrate semiquantitative stiffness differences between inflamed and adjacent tissue [7,8].

**Figure 4:**
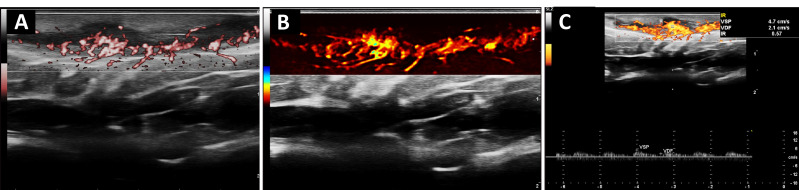
Doppler imaging was performed using the same acquisition parameters as those described in Figure 2 (high-frequency linear probe at 12.5 MHz; PRF of 480 Hz optimized for low-velocity flow detection). (**A**) Power Doppler imaging demonstrates a hypoechoic inflammatory sinus tract (subcutaneous tunnel) with prominent intralesional and perilesional vascular signals, consistent with active inflammatory involvement. (**B**) Advanced Doppler modality (Microvascular/Enhanced Doppler highlights dense low-velocity vascular networks within the tract wall, reflecting inflammatory angiogenesis and microvascular remodeling. (**C**) Spectral Doppler interrogation of an intralesional vessel shows a low-resistance arterial waveform (VSP 4.7 cm/s; VDF 2.1 cm/s; RI 0.57), supporting active inflammatory hyperemia. These multimodal Doppler findings provide structural and hemodynamic characterization of sinus tract activity, consistent with Doppler-based inflammatory biomarkers described in HS [4,5,6].

**Figure 5:**
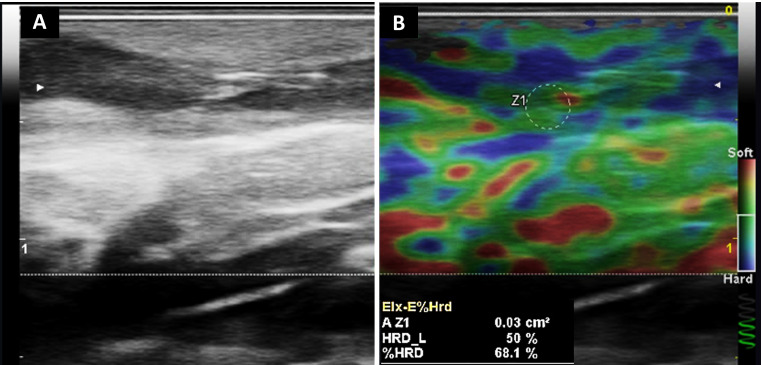
Structural and biomechanical characterization of the same inflammatory sinus tract previously illustrated in Figure 4. (**A**) B-mode ultrasound demonstrates a hypoechoic dermo-subcutaneous tunnel with architectural distortion of the surrounding tissue. (**B**) Strain elastography of the identical lesion reveals heterogeneous stiffness distribution within the tract wall. Elastography was performed using light repetitive manual compression under real-time quality control, monitored by the calibrated spiral indicator. Three consecutive compression cycles were obtained for each region. Semiquantitative measurements were acquired from four regions of interest (ROIs; 0.03 cm^2^ each), including the tract wall (Z1–Z2) and adjacent reference tissue without lesion (Z3–Z4) for internal comparison at comparable depths within the elastographic field, with ROI placement guided by the corresponding B-mode anatomical reference and avoiding inclusion of fluid components or acoustic artifacts. The altered tract wall demonstrated variable hard component values (%HRD), ranging from 68.1% to 100%, whereas adjacent reference tissue showed substantially lower stiffness values (e.g., 0–13.6% HRD). Strain ratios (Elx2/1, Elx3/1, Elx4/1) ranged from 2.42 to 5.09, confirming relative biomechanical heterogeneity between inflamed and non-involved tissue. These findings illustrate reproducible semiquantitative stiffness variation across repeated compression cycles and support strain elastography as a complementary imaging biomarker for inflammatory remodeling in HS [7].

**Figure 6:**
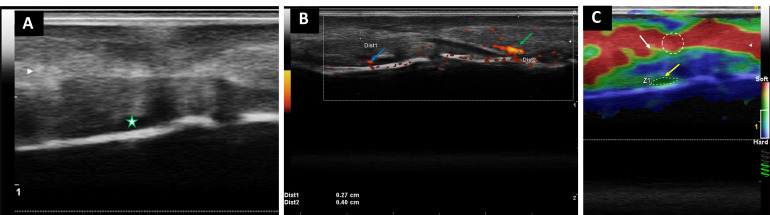
Multimodal ultrasound assessment of palmar plate enthesitis at the right third distal interphalangeal (DIP) joint. (**A**) B-mode ultrasound of the volar aspect of the right third DIP joint demonstrates hypoechoic thickening at the insertional enthesis of the palmar plate with adjacent anechoic effusion (star), consistent with ultrasound-defined enthesitis according to OMERACT criteria. (**B**) Power Doppler acquisition was performed with the same technical parameters as those described in Figure 2. Power Doppler imaging reveals a vascular signal at the insertional enthesis of the palmar plate (blue arrow) and at the deep digital flexor tendon insertion (green arrow), supporting active enthesitis and flexor tendon–related inflammatory involvement. (**C**) Strain elastography demonstrates heterogeneous stiffness distribution at the insertional enthesis and adjacent perientheseal tissue. Elastography was acquired using light repetitive manual compression with real-time quality control monitored by the calibrated green spiral indicator. Three consecutive compression cycles were performed, and semiquantitative measurements were obtained from regions of interest (ROIs; 0.02–0.03 cm^2^) positioned over the insertional enthesis and adjacent reference tissue for internal comparison at comparable depths within the elastographic field to ensure consistent semiquantitative comparison, with ROI placement guided by the corresponding B-mode anatomical reference and avoiding inclusion of fluid components or acoustic artifacts. The insertional enthesis exhibited hard component values ranging from 79.9% (white arrow, perientheseal extension) to 100% (yellow arrow, insertional enthesis) (%HRD), whereas adjacent structurally normal reference tissue demonstrated markedly lower stiffness (4.5% HRD). Strain ratios ranged from 0.42 to 2.15, indicating biomechanical heterogeneity consistent with active inflammatory enthesitis. These findings are consistent with ultrasound-defined digital enthesitis and flexor tendon–related inflammatory involvement, overlapping with domains incorporated into the GLobal OMERACT Ultrasound DActylitis Score (GLOUDAS) framework [9]. Articular inflammatory manifestations have also been described in association with HS [10].

## Data Availability

The data presented in this study are available on request from the corresponding author due to patient privacy and ethical restrictions related to identifiable clinical imaging data.
